# Application of Various Optical and Electrochemical Nanobiosensors for Detecting Cancer Antigen 125 (CA-125): A Review

**DOI:** 10.3390/bios13010099

**Published:** 2023-01-06

**Authors:** Mehrab Pourmadadi, Ali Moammeri, Amin Shamsabadipour, Yasamin Farahanian Moghaddam, Abbas Rahdar, Sadanand Pandey

**Affiliations:** 1 School of Chemical Engineering, College of Engineering, University of Tehran, Tehran 11155-4563, Iran; 2 Department of Physics, University of Zabol, Zabol 98613-35856, Iran; 3 Department of Chemistry, College of Natural Science, Yeungnam University, 280 Daehak-Ro, Gyeongsan 38541, Republic of Korea

**Keywords:** electrochemical nanobiosensors, optical nanobiosensors, ovarian cancer, cancer antigen 125 detection

## Abstract

Nowadays, diagnosing early-stage cancers can be vital for saving patients and dramatically decreases mortality rates. Therefore, specificity and sensitivity in the detection of cancer antigens should be elaborately ensured. Some early-stage cancers can be diagnosed via detecting the cancer antigen CA-125, such as ovarian cancer, and required treatments can be applied more efficiently. Thus, detection of CA-125 by employing various optical or electrochemical biosensors is a preliminary and crucial step to treating cancers. In this review, a diverse range of optical and electrochemical means of detecting CA-125 are reviewed. Furthermore, an applicable comparison of their performance and sensitivity is provided, several commercial detection kits are investigated, and their applications are compared and discussed to determine whether they are applicable and accurate enough.

## 1. Introduction

Ovarian cancer (OC) is the main cause of death in women with gynecologic malignancies, and it is a common malady in the female reproductive system. It also ranks fifth among the most common causes of mortality in women associated with cancers. Over the last few decades, despite all the medical advances in cancer therapy, the survival rate for OC has not significantly progressed. Moreover, this disease has been classified as a serious threat to women over the age of 50 due to its high fatality-to-cause ratio. To reduce the mortal severity of this cancer, it is crucial to have early-stage detection [1,2,3,4].

There is a vital need to develop OC-related biomarkers to promote prognostication of the disease and lessen its dismal mortality rate. Cancer antigen 125 (CA-125) is a well-known oncomarker in OC that has been widely used in the last three decades. CA-125 is a high-molecular-weight glycoprotein (>200 kDa) generated by normal cells in adult tissues derived from coelomic and Müllerian epithelia. CA-125 levels of less than 35 U/mL in the human body are acceptable for normal cells. Women with advanced stages of OC often show an elevated level of CA-125 [5,6,7]. In the past few years, several studies have been conducted to prove the usefulness of this antigen for the diagnosis and prognosis of OC. Therefore, the FDA (the US Food and Drug Administration) officially approved the CA-125 test for the early-stage detection of OC [8,9].

The complex instrumentations and intricate protocols of conventional methods used for measuring cancer biomarkers require researchers to find a selective and sensitive detection technique for rapid diagnosis. Despite various improvements in existing analytical techniques, such as enzyme-linked immunosorbent assays (ELISAs), the mass spectrometric immunoassay, radioimmunoassay, and fluorescent spectrometry, they suffer from low sensitivity and selectivity along with a high cost. Thus, it is essential to monitor and quantify oncomarkers through a point-of-care system [10,11,12].

In recent decades, biosensors have been proposed as the best alternative technology to conventional detection methods in clinical applications and point-of-care diagnosis. Excellent selectivity and sensitivity, portability, and minimum requirements for sample pretreatment are the advantages biosensors have over conventional diagnosis techniques [13,14,15]. Integration of nanomaterials in the structure of biosensors provides sensitive, specific, and fast-response sensors for special functions. In recent years, researchers have proposed numerous biosensors incorporated with several nanomaterials and biomaterials with different sensing approaches to detect and monitor cancer biomarkers [16,17,18]. Miscellaneous nanocomposites based on various nanomaterials have been developed as transducer materials and support for the immobilization of biomolecules. Gold nanoparticles (Au NPs), graphene and its derivatives, carbon-based nanomaterials, and magnetic NPs have always been the main candidates in designing biosensors with better sensitivity and conductivity. In most cases, integrating nanomaterials with biorecognition elements, including enzymes, antibodies, and aptamers, has brought promising results in selectivity and sensitivity. Biomaterials have been widely used as bioreceptors to detect cancer-specific biomarkers. The conjugation of nanomaterials with bioreceptors has been conducive to the development of advanced electrochemical and optical biosensors for the detection of cancer biomarkers, such as CA-125 [19,20,21,22,23].

Optical and electrochemical methods are the two major analytical strategies that have been widely used in biosensors. Optical biosensors detect the optical signals produced by the interaction between a bioreceptor and the optical field in the presence or absence of analytes. However, chemiluminescence (CL)- and electrochemiluminescence (ECL)-based methods harness the optical signal produced by a chemical reaction for further measurements [24,25,26]. Furthermore, Raman spectroscopy, a putative method for the optical detection of cancer biomarkers, uses the scattering of incident light passed through a sample to measure the vibrational energy of molecules and the refractive index [27,28]. Electrochemical methods rely on turning biochemical events into electrical signals produced by changes in current, resistance, or capacitance on the surface of an electrode. For instance, electrical impedance spectroscopy measures the resistance on the electrode’s surface, whereas voltammetry-based biosensors measure the electrical current as a function of applied potential [29,30,31].

This work provides a retrospective review of optical and electrochemical biosensors, such as fluorescent biosensors, chemiluminescence (CL) and electrochemiluminescence (ECL) biosensors, surface-enhanced Raman scattering (SERS), and surface plasmon resonance (SPR) biosensors, colorimetric biosensors, electrical impedance spectroscopy (EIS) and voltammetric biosensors, and other types of biosensors for the detection and quantification of CA-125 oncomarkers.

## 2. Optical Biosensing of CA-125

**Figure 1:**
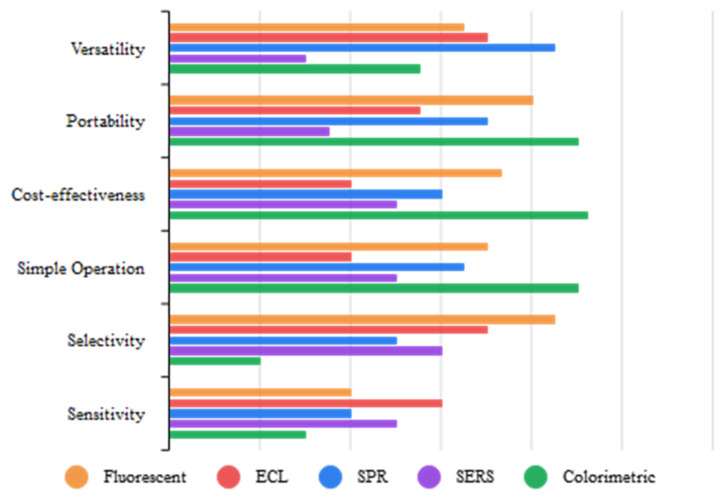
Comparison of optical methods.

Optical biosensors are analytical tools with optical detection systems that have been widely used in medicine, biomedical studies, the food industry, environmental monitoring, and pharmaceutical sciences. They consist of a biological recognition element integrated with an optical transducer to transmit changes in light responses and intensity in chemical and biochemical interactions between the measured substance and the probe. To be more specific, they excite analytes through a specific light wavelength to elevate the analytes’ energy levels. When they return to their normal level of energy, the surplus energy is freed in the form of photons. Owing to the existing optical approaches to detect and measure analytes, including luminescence and fluorescence, plasmon resonance, Raman scattering, ECL, and colorimetric methods, different classes of optical biosensors have been developed [32,33]. Due to their real-time and direct monitoring along with their ability to carry out multiplexed detection of many analytes, optical biosensors have replaced conventional methods of detecting and measuring CA-125 oncomarkers in biomedical research [34,35]. Figure 1 summarizes the advantages and limitations of optical methods used for biosensing cancer biomarker detection. Table 1 reviews previous studies on the application of optical biosensors for CA-125 determination.

### 2.1. Fluorescence-Based Biosensors

Fluorescence has become the leading optical method in biosensing owing to its low cost, simple operation, excellent selectivity, and high efficiency. The variations in the fluorescent characteristics of a bioreceptor, as a consequence of its interaction with an analyte, lead to its determination and detection [74,75]. Therefore, taking into account all these advantages and principles, researchers have proposed numerous fluorescent biosensors for the detection of CA-125 [31,32].

**Figure 2:**
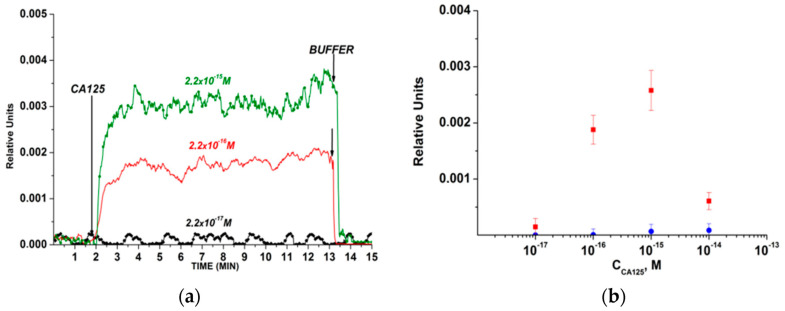
The outcomes acquired from the detection of CA-125 protein in buffer solution while employing a silicon-on-insulator (SOI) nanowire biosensor with covalently immobilized antibodies: (**a**) common sensorgrams acquired upon assessment of solutions with diverse concentrations of the target protein; (**b**) dependencies of the level of the biosensor signal on the concentration of CA-125 in buffer solution [76].

For example, Abou-Omar et al. [44] provided a rapid, accurate, and sensitive nano-optical sensor based on a thin sol–gel film incorporating Au NPs for CA-125 detection in serum samples from normal women and patients diagnosed with OC. In this work, Au NPs were covered by a Schiff base ligand and then fixed into a sol–gel matrix to assess their optical properties using UV–vis spectrophotometry. Upon the addition of the cancer antigen, the absorbance intensity decreased. Then, the fluorescence emission spectra of this gold–Schiff base complex-doped sol–gel were investigated before and after the addition of CA-125. According to the results, the fabricated nano-optical sensor revealed a linearity of 2.0 to 127.0 U/mL with an LOD of 1.45 U/mL. In another study, Malsagova et al. [76] developed a silicon-on-insulator (SOI) nanowire biosensor and immobilized antibodies covalently on it to detect the CA-125 cancer antigen (Figure 2). They concluded that as the protein concentration increases, the signal of the biosensor, which demonstrates the interaction among antibodies and CA-125, intensifies. Based on the results, they determined the minimum detectable concentration of the protein equals 1.5 × 10^−16^ M.

In another work conducted by Bahari et al. [41], they measured CA-125 and CA15-3 tumor markers through an efficient immunosensor by applying the sensitivity of a fluorescence method and the great specificity of the synthesized magnetic molecularly imprinted polymers (MMIPs). In this study, they used noble Cd nanoclusters (NCs) and Ni NCs as effective and economic emitters along with magnetic graphene oxide (GO–Fe_3_O_4_) as a platform to support MMIP. The results revealed that by increasing concentrations of CA-125 and CA15-3, the fluorescence strength of the Ni NCs and Cd NCs was elevated. The fabricated optical sensor showed excellent properties in terms of linearity range (0.0005–40 U/mL) and LOD (50 μU/mL). This work claims that this imprinted immunosensor can be used as a clinical device for checking for breast cancer and OC.

Xu et al. [43] constructed a double-color biosensor based on aptamers for the simultaneous determination of the carcinoembryonic antigen (CEA) and CA-125. They used salt-provoked Au NPs’ mass to light up the fluorescence of a dual-color DNA–silver NCs-aptamer (DNA-Ag NCs-Apt). Their color-based system comprised red-producing DNA-Ag NCs with the aptamer (rDNA1-AgNCs-Apt1) and green-emitting DNA-Ag NCs beside the CEA aptamer (gDNA2-AgNCs-Apt2). By applying this fluorescence aptasensor, an LOD of 0.015 U/mL was achieved for CA-125.

#### Fluorescence Resonance Energy Transfer (FRET)-Based Biosensors

This technique depends on nonradiative energy transmission between two fluorescent materials, a “donor” fluorophore to an “acceptor” fluorophore. Owing to its good sensitivity, fast sample analysis, and low background signal, it has been widely used as an important tool used to monitor protein interactions in biological studies [45].

Omer and colleagues fabricated an ultrasensitive optical biosensor made up of carbon quantum dots (CQDs) for CA-125 detection in the early malignant stage. Their method relies on the quenching mechanism upon the interaction between CQDs and CA-125. The performance of the proposed optical sensor was remarkable due to its low LOD of 0.66 U/mL within the concentration range of 0.01 to 129 U/mL [47].

### 2.2. Chemiluminescence-Based Biosensor

In this method, a chemical reaction between a biological recognition element and an analyte gives rise to produce a luminescence emission of light. By employing this energy, generated by returning an excited molecule to its ground state, researchers have proposed many CL biosensors as the most sensitive optical method [77,78]. Owing to their incredible sensitivity, simple instrumentation, and broad dynamic range, they have been widely used for the detection of various oncomarkers [79]. Several CL biosensors have been familiarized as a sensitive means for the quantification of CA-125 oncomarkers, some of which are presented in Table 1.

Al-Ogaidi et al. [49] synthesized graphene quantum dots (GQDs) for chemiluminescent immune-chip fabrication. They transferred chemiluminescence resonance energy from CL reagents to GQDs. The proposed immunosensor could detect CA-125 at a concentration of 0.05 U/mL with a linear concentration range of 0.1 to 600 U/mL.

### 2.3. Electrochemiluminescence-Based Biosensors

This method uses an electrochemical process to trigger CL by which the radiated light is sensed in the presence of the desired voltage. Compared to other optical techniques, this method does not require an outside light source; thus, its major benefit over other techniques is the reduction in background signal. Moreover, ECL-based biosensors take advantage of the low cost of electrochemistry together with the sensitivity of luminescence [80].

Babamiri et al. [55] proposed an ultrasensitive immunosensor for the simultaneous measurement of cancer antigen 153 (CA15-3) and CA-125 tumor markers. They used dendrimer-sulfanilic acid-Ru(bpy)_3_^2+^ and polyamidoamine dendrimer-QDs along with Fe_3_O_4_–SiO_2_ as an immunosensing platform and the carrier for reactants generating ECL. Their results reveal that the fabricated ECL immunosensor had an LOD of 0.1 µU/mL in the concentration range 1 µU/mL to 1 U/mL. The performance of the biosensor was evaluated in the human serum sample. According to the results, there was good harmony with the results obtained by the ELISA method.

In another study, Yin and colleagues [57] designed a near-infrared (NIR) ECL immunosensor with the core/shell AgInS_2_/ZnS nanocrystals (NCRs). By oxidizing the synthesized NCRs, both the monodispersed AgInS_2_/ZnS NCRs and the surface-confined AgInS_2_/ZnS NCRs formed sandwich-typed immuno-complexes with CA-125. Under physiological conditions, the designed immunoassay showed a low LOD (1 × 10^−6^ U/mL) in a broad linear range (5 × 10^−6^–5 × 10^−3^ U/mL), and can thus eventually be used as an effective tool for CA-125 determination in the early diagnosis of OC.

### 2.4. Surface Plasmon Resonance (SPR)-Based Biosensor

This label-free optical method utilizes the affinity interaction between a probe and a target to increase the refractive indicator at the surface of SPR sensors. By observing changes in the refractive index, the reaction can be measured. This method provides researchers with a rapid and label-free tool to detect oncomarkers in clinical diagnosis [81,82].

Szymańska and coworkers [65] used a thiol-modified gold surface for CA-125 detection via its antibody. In this work, the linear range was well-suited for use to determine the analyte in blood serum (2.2–150 U/mL). In the end, the designed sensor was successfully tested in real samples from patients diagnosed with OC.

Rebelo et al. [66] developed an electrochemical sensor and an SPR optical sensor based on pyrrole (Py) electropolymerization on a Au screen-printed electrode (SPE). The SPR sensor provided a high-quality analysis of CA-125 while it was interacting with MIP. The linear range and LOD of the SPR sensor for CA-125 determination were 0.1–300 U/mL and 0.1 U/mL, respectively.

### 2.5. Surface-Enhanced Raman Scattering (SERS)-Based Biosensor

This analytical method provides an enhanced Raman signal of molecules when they come to contact with nanostructured metallic surfaces. SERS-based biosensors have increasingly progressed in mapping and detecting oncomarkers. High resolutions and the possibility of multiplexed diagnosis make them a favorable tool for the simultaneous determination of several targets [83,84,85].

Tunc et al. [69] designed a sensing platform based on a self-assembled monolayer of Au to detect and determine the CA-125 biomarker. They localized highly enhanced electromagnetic fields near Au NPs and recorded CA-125 antibody and antigen couples. According to the results, there were major changes before and after CA-125 antibody–antigen bioconjugation in the SERS spectra and hot-spot SERS mapping, proving CA-125 binding.

### 2.6. Colorimetric Biosensor

Compared to previous optical biosensing methods, this technique is a simple method to detect a target by visual changes in color induced by the bioconjugation between the probe and the analyte. The low cost and simple instrumentation are the major advantages of this method, which make it promising for cheap and portable detection of oncomarkers [86,87].

Hosu et al. [72] developed a colorimetric smartphone-enabled immunosensor based on a 3D nitrocellulose membrane and Au NPs for sandwich immobilization of the primary and secondary antibodies, respectively. The formation of an antibody–Au NPs complex caused Ag in an enhancer solution to be deposited and form Au/Ag nanocomposites in different gray colors based on the concentration of CA-125. They used an eight-megapixel camera for smartphones to determine image pixel intensity. The designed sensor revealed high sensitivity, and its LOD was 30 U/mL.

### 2.7. Brief Overview of Optical CA-125 Biosensors

In general, there have been various optical methods, including fluorescence, FRET, CL, ECL, SERS, SPR, and colorimetric, for CA-125 determination. The notable merits of optical biosensors, such as their great sensitivity, excellent selectivity, and easy instrumentation, make them a great alternative to conventional methods in the prognostication of OC. Photoluminescence [48] and plasmon resonance scattering (PRS) [71] are other techniques reported in recent studies, achieving LODs of 0.07 ng/mL and 0.4 U/mL, respectively, for CA-125 detection (Table 1). Fluorescent and ECL-based biosensors are the most studied tool in the detection of CA-125 biomarkers due to their excellent characteristics, such as their low cost and high sensitivity, along with great selectivity. Among existing optical methods, fluorescence, FRET, and ECL have the best performances in the determination of CA-125 due to their low LOD. According to the LODs reported in previous works, sandwich nano-immunosensors and aptasensors demonstrate the highest sensitivity toward CA-125 determination. Interestingly, in a study conducted by Hamd-Ghadareh et al. [45], an antibody–aptamer sandwich fluorescent immunosensor based on PAMAM-dendrimers/Au NPs was utilized for CA-125 detection. Their designed nanobiosensor achieved an LOD of 0.5 fg/mL. Moreover, some researchers have used multiplexed detection techniques for simultaneous optical measurement of CA-125 with other cancer biomarkers. Nano-biochips, disposable paper-based devices, and smartphone-based immunoassays are novel and attractive methods used for CA-125 detection (Table 1). Despite all the advances in optical biosensors, there is an essential need to develop optical methods for point-of-care and commercial detection.

## 3. Electrochemical Biosensors

**Figure 3:**
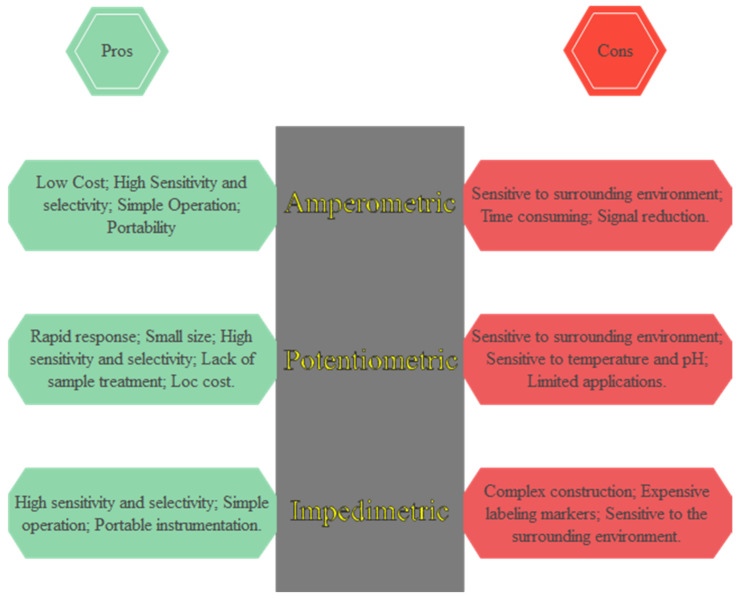
Comparison of electrochemical methods.

Biosensors translate biological parameters into electrical currents. An electrode is a critical component in this sort of sensor, serving as a firm foundation for biomolecule immobilization and electron flow. Synergic effects are enabled by many nanomaterials with large surface areas, which improve loading capacity and reactant mass transit to achieve high analytical sensitivity. Electrochemical biosensors are analytical tools that turn biochemical events into electrical signals. Enzyme–substrate reactions and antigen–antibody interactions are two examples of these events [88]. Figure 3 shows the pros and cons of electrochemical methods used for biosensing cancer biomarkers.

### 3.1. Electrical Impedance Spectroscopy-Based CA-125

When analyzing the interfacial characteristics of surface-modified electrodes, electrochemical impedance spectroscopy (EIS) is a useful technique for measuring the impeded flow of ions across solutions, interfaces, and coatings. The EIS approach is frequently used to investigate the kinetics of the electrode, the behavior of adsorption, and the connection of biomolecules with the electrode surface [89]. Electrochemical impedance is typically analyzed by employing an AC potential of diverse frequencies to an electrochemical cell and quantifying the current which passes from the cell. The voltage signal as a result delays the current signal by a specific phase angle. The Laplace transformation converts the time-related signal Z(τ) to the frequency-related signal Z_0_, resulting in a complex number that may be calculated as follows [90].

Z(ϖ) = ϑ/I = ϑ_0_ cos(wτ)/I_0_ cos(wτ + ψ) = Z_0_ exp(Iψ) = Z_0_ (cos ψ + I sin ψ)

In the above correlation, ϖ indicates the frequency of the utilized potential, ψ stands for the phase angle, ϑ is representing the alternating voltage in which ϑ_0_ is the amplitude of the alternating voltage, and it demonstrates the alternating current in which I_0_ is the amplitude of the alternating current [90].

Z^2^ = Z_im_^2^ + Z_real_^2^

**Figure 4:**
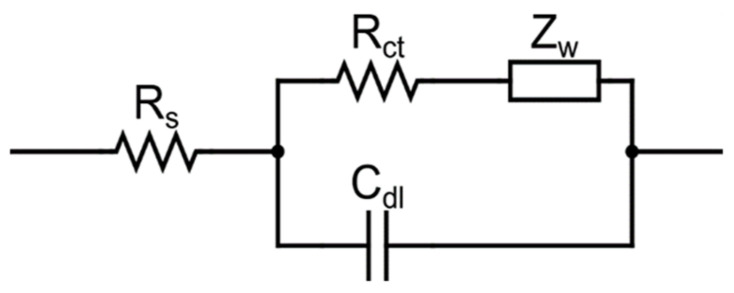
Simulating electrode–solution interface (C_dl_).

The Nyquist plot, on the other hand, illustrates the real and imaginary elements of impedance on the X and Y axes, respectively. It can be simulated by a similar circuit (Randles circuit) including the solution resistance (RS), resistance employed for charge transferring (R_ct_), Warburg impedance (Z_W_), and double-layer capacitance (C_dl_) (Figure 4). The value of R_ct_ is determined by the semicircle diameter of the EIS spectrum, which reveals the kinetics of electron transport at the electrode interface for the redox probe. Furthermore, R_S_ and Warburg impedance (Z_W_) characterize the diffusion of the applied redox probe and the bulk properties of the electrolyte solution, respectively. Z_W_ describes the electrical response at the electrode and may be calculated using the Nyquist plot. It is defined as the intercept of a line with a slope of 45 degrees. An analogous electrical circuit for an electrode can also be created (Figure 4). A label-free detection technology is commonly used by researchers to identify cancer cells.

The POISED-5 instrument was created to combine the benefits of using a limited number of discrete frequencies with a simplified impedance assessment while acquiring an analog waveform resolution comparable to that achieved with existing commercial frequency response analyzer (FRA) equipment. The tool was employed to quantify CA-125, the gold standard biomarker for the progressed stage of OC diagnosis and deterioration after the chemotherapy process, after indicating the system’s efficacy by measuring voltages that exceeded (low impedance) or undershot (high impedance) threshold values employing solid condition “standards”. The technology was created by immobilizing anti-CA-125 antibodies on a screen-printed graphene biosensor and detecting CA-125 protein using a five-frequency EIS technique [91].

The presented study demonstrates a selective, sensitive, cost-effective, and fast technique for CA-125 measurement by employing the quenching capability of Au NPs coated with a Schiff base ligand laid in a fine sol–gel film. Bizarre characteristics, including molecule-like HOMO-LUMO energy gaps and single-electron charging, were observed when Au NPs were preserved by a layer of Schiff base ligands, allowing their usage in chemical and optical sensing [44].

The benefits of a new CA-125 electrochemical and optical biosensor built by electropolymerization on gold SPEs (Au-SPE) and SPR gold sensors employing a Py monomer are compared in this work [66]. The application of Py to create highly selective printed materials for a protein improved the performance of the biosensor. CA-125 was covalently linked to the previously cysteamine-treated Au-SPE film [20].

### 3.2. Voltammetry-Based CA-125

Voltammetry is a class of amperometric methods that measure electrical current based on an applied potential. DPV is one of the most frequently used voltammetric methods due to its great sensitivity and rapidity. This approach includes superimposing a series of fixed-amplitude electrochemical pulses (10–100 mV) over a steadily increasing base potential, and then measuring and graphing the resultant current difference vs. the base potential. The analyte concentration is obtained by employing the outcome of the peak current. DPV technology has been used to investigate several approaches for detecting early cancer and examining how cancer-related drugs operate. As a result, it is fair to believe that a multiplexed marker identification method can be a remarkable diagnostic device for cancer detection in clinical applications. Multianalyte diagnosis can provide fast, selective, sensitive, and cost-effective detections. Some measurements have been taken to identify several cancer biomarkers at the same time. The authors devised disposable two-throughput immunoelectron arrays for the coincidental identification of the cancer antigens CA 19-9 and CA-125 [90,92].

### 3.3. PEC Electrochemical CA-125

Photoelectrochemical (PEC) detection provides excellent sensitivity, rapid detection, a cheap cost, and simple equipment. It is used to analyze food safety, detect biological agents and pharmaceuticals, and monitor the environment. PEC sensors are made of titanium dioxide (TiO_2_), cadmium sulfide (CdS), zinc oxide (ZnO), and copper(I) oxide (Cu_2_O), which have increased worries about their photoactivity. Some techniques have been employed to enhance the performance of photoelectric substances. Photoelectric conversion efficacy can be enhanced by designing, doping, and merging semiconductors with metal or other semiconductors [93]. A Schottky connection was formed between AuNPs and gallium nitride (GaN) by growing AuNPs in situ on the surface of GaN, and afterward, etching them on the preferred size of diameter with H_2_O_2_. To improve migration efficacy, photogenerated electrons from GaN can be captured and transferred by AuNPs. The separation of the electron–hole pairs ameliorates the photoelectric operation of the system. By altering the size of the AuNPs, the Fermi energy level of the AuNPs and the charge transfer efficacy of Au NPs/GaN may be altered. Then, utilizing a Au NPs/GaN Schottky photoelectrode, a novel PEC aptasensor for the detection of the epithelial OC marker CA-125 was constructed. This approach demonstrated good sensitivity, specificity, and effectiveness in the detection of CA-125 serum. Capturing electrons from GaN and transferring them to Au NPs considerably boosted the PEC signal of the tool. Moreover, the aptamer of CA-125 on the Au NPs was modified by Au-S interaction. When the aptamer bonds to the target, the protein inhibits the system’s photoelectron transfer pathway, causing the photocurrent to decline. Based on the link between the system’s photocurrent and CA-125 concentration, a sensitive PEC sensor can be constructed to detect CA-125. The suggested technique has been employed with high efficacy to detect CA-125 in human serum [94,95].

### 3.4. Other Electrochemical CA-125

In electrochemical immuno-biosensing, the antigen–antibody interaction generates a quantifiable electrical signal that is based on electrochemical principles [96]. Electrical immuno-biosensors are classified as voltammetric, amperometric, impedance, or capacitive sensors based on the electrical signal detected during biomolecule contact. The specific advantages of capacitive electrical immuno-biosensing are the simplicity of the detection technique, the availability of flexibility in the sensitivity settings, and the low-power-consumption performance [87,97]. The immuno-biosensing on the microfluidic platform makes electrochemical biosensing assays portable, making it straightforward to apply this sensing mechanism in point-of-care devices. Microfluidics can identify many biomarkers from the same sample (multiplex assay), minimizing the risk of inaccurate illness detection. The microfluidic platform is used to separate the targeted biomolecules for sensing, which reduces interference from outside signals and improves the signal-to-noise ratio.

The demand for innovative, low-cost CA-125 detection methods has significantly grown due to the increasing OC risk recurrence and the shortcomings of the present detection processes. Furthermore, to create a novel technology for quick and precise CA-125 detection, a sensitivity analysis of CA-125 detection was conducted in this experiment.

The shear created by microfluidic flow during antigen–antibody interaction, as well as the change in capacitance with biofluid static depth and microfluidic flow (CA-125 antigen solution), was discussed in this study. This study of sensitivity variation will be useful to future researchers of capacitive biosensing on microfluidic devices [98].

As mentioned in Table 1, H_2_O_2_ is produced as a byproduct of oxidative enzymes. Using an accurate method of detection is necessary. Detecting tumor biomarkers, such as H_2_O_2_, aids in cancer diagnosis. The concentration of CA-125 will increase H_2_O_2_; hence, we detected H_2_O_2_ [99].

HCI-doped polyaniline, chitosan hydrochloride composite, and Ag-Co304 nanosheets were also employed as high-antibody immunosensors [100].

Due to their structure and combination of metal ions and organic ligands, metal–organic framework (MOF) nanoparticles are beneficial in biology [101]. A carbon nanotube (CNT) was produced using inorganic chemistry (MOF-808) 11ith MOF-808 and CNTs. CA-125 was created using the protein-friendly, high-surface-area MOF-808’s electrocatalytic activity (CA-125). Electrochemistry was increased by MOF-808/CNT. Electrochemistry using a glassy carbon electrode (GCE) and MOF-808/CNT for label-free immunosensor Streptavidin-enhanced MOF-808 antibodies was carried out, and the immunosensor’s linear range was revealed to be 0.0010.1 to 0.130 ng/mL (S/N 3) [102]. Some information about the performances of various electrochemical biosensors for the detection of CA-125 has been provided in Table 2 and Table 3.

### 3.5. Brief Overview of Electrochemical CA-125

Numerous electrochemical properties and phenomena have been used as a basis for the detection of CA-125, including but not limited to the electrical impedance spectrum, voltage–current curves, and the use of photoelectrons as proxies for measuring the optical spectra. Biosensor performance analysis and construction rely heavily on sensitivity research [90]. Nanoparticles can be used to diagnose and image cancer. Molecular markers on cancer cells can be found with metal nanostructures, carbon-based nanoparticles, polymer hybrid nanomaterials, and antibody-functionalized quantum dots [103]. Few studies have examined how microfluidic flow affects capacitance sensor sensitivity in biosensing. There is no information on the change in capacitance of interdigitated electrodes during CA-125 detection in the presence of microfluidic flow and static drop [98]. All these methods share the common principle of converting the proliferation of CA-125 to a relationship between the electric potential and current. In EIS, specific markers are found and bound to the detector, which then undergoes spectroscopy to detect the levels of the reactants. In the case of CA-125, screen-printed graphene [91] and a sol–gel film containing coated Au NPs [44] have been used to varying degrees of success. Voltage–current curves are elicited by applying special voltage sweeps to the reactants and measuring the voltage in a technique known as voltammetry. Here, electrode design poses a challenge as well, with materials such as graphene providing desirable properties when processed [90]. Electrical measurements of photochemical phenomena benefit from the recent advances in semiconductors, with new materials showing potential for new means of detecting CA-125 [98]. Other exemplary works discussed here additionally use microfluidic chips and nanostructures as promising research venues in this field [105].

## 4. Comparison of the Performance of CA-125

**Figure 5:**
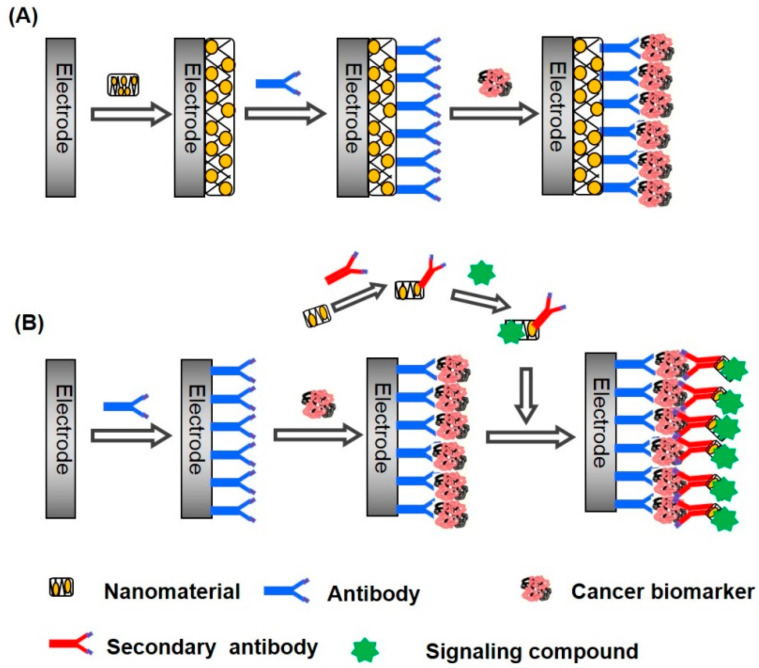
Employing nanomaterials for surface functionalization (**A**) with a signaling label (**B**) in constructing electrochemical biosensors for cancer biomarkers [119].

In the current review, plenty of optical and electrochemical sensors used for the detection of CA-125 have been examined. The outcomes revealed good sensitivity with a low LOD, great selectivity, and repeatability for detecting the cancer antigen CA-125. The main benefits and drawbacks of those sensors for the detection of cancer antigen CA-125 are represented in Table 4. Furthermore, surface modification, functionalization, and ligand structures can play a key role in the efficiency of both diverse electrochemical and optical biosensors for detecting antigen CA-125 and other cancer biomarkers (Figure 5). For instance, in the case of electrochemical biosensing of CA-125, numerous works have been recently carried out employing and modifying Au NPs, such as by immobilizing them or coating them with organic polymers and inorganic elements to fabricate more efficient platforms, which can improve the biological loading of CA-125 and enhancing antigen–antibody conjugation [102,105,106,107,108]. Additionally, in the case of the optical detection of CA-125, we are observing more diversity in respect of structural functionalization, especially in some recent studies, which were implemented using an aptamer to modify the biosensing process. Heidari et al. [109] developed a carbon dot (CD) probe with aptamer conjugation and implemented modification and hybridization on an aptamer with CA-125 via ameliorated fluorescent bioimaging. Therefore, optical sensors are can be applied more efficiently in both detection and selection than electrochemical biosensors due to the specific functionalization that has been carried out.

It is also worth mentioning that novel biosensors, both electrochemical and optical ones, are developed with high accuracy and selectivity toward malignant tumors versus benign ones in serum based on the characteristic features of the malignant tumors in comparison with benign tumors and the higher affinity of the functionalized ligands with the malignant tumors [110,111,112]. Moreover, there are some recently developed and advanced biosensors that can identify malignant tumors with high selectivity and sensitivity merely based on their special nodular and stellate shapes [113,114].

**Table 4:** Comparison of benefits and drawbacks of optical and electrochemical sensors for detecting CA-125 antigen.

Principle	Benefits	Drawbacks	References
Optical biosensors	- Real-time; - Specific detection of CA-125 - No need for warm up processing - Quantitative - High selectivity - Low LOD	- Rare clinical employment - Typically expensive - Difficult construction - Restrictions in detecting multiple analytes	[18,115,116]
Electrochemical biosensors	- High sensitivity - Convenient and quick - Simple miniaturization - Recyclable - Quantitative - Cost-efficient	- Rare clinical application - Difficult to control sensing electrode at high currents - False-positive results initiated by electrolytes - Hard surface control of sensing electrode at high currents.	[10,117,118]

## 5. Comparison of CA-125 Commercial Detection Kits

**Figure 6:**
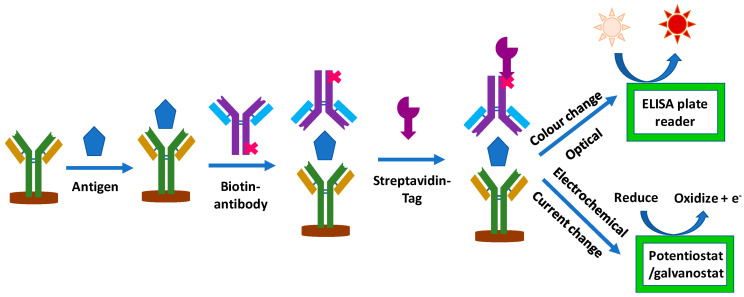
Schematic of immunosensor operating and detection mechanism [126].

Currently, most commercial CA-125 detection kits are based on colorimetric methods such as ELISA (Table 5), and some recent developments have been implemented to improve electrochemical ELISA-based immunoassays’ detection of cancer biomarkers (Figure 6). Jiang et al. [15] detected five serum protein markers including CA-125 by employing the antibody technique for OC. In another study, Nawaz et al. [120] performed a comparative investigation between IMMULITE and ELISA for the detection of CA-125 (OC) in a diverse age range by employing ELISA commercial kits. They carried out their experiments on 80 patients with OC and 6 healthy persons. ELISA detected CA-125 in 57 individuals, while CA-125 was detected in 64 patients with IMMULITE; therefore, they concluded that although the sensitivity of IMMULITE assays is higher, they are not widely available or cost-effective. Therefore, in these circumstances, employing ELISA commercial kits can be applicable.

It is also worth considering that biosensors for the detection of CA-125, both electrochemical and optical ones, are mostly recyclable and more cost-efficient than conventional detection techniques such as ELISA kits [121,122,123]. Furthermore, based on previous reports, most biosensors for sensing CA-125 have an LOD of lower than 1.45 U/mL with a sensitivity of higher than 97% and a specificity of higher than 94% [124], while ELISA kits achieved values higher than 2 U/mL [125]. In respect of their selectivity in sensing CA-125, biosensors can be applied effectively with higher selectivity than ELISA kits [31].

## 6. Conclusions and Future Perspectives

In recent years, both optical and electrochemical detecting procedures have progressed in the detection of CA-125. These developments led to an enhanced detection of CA-125 with higher sensitivity and specificity. The outcomes of diverse optical detection methods revealed their high sensitivity, excellent selectivity, and simple instrumentation, which indicate this technique is an applicable approach to the detection of CA-125. Furthermore, the same results of various electrochemical detection methods demonstrated high sensitivity, simple miniaturization, recyclability, and cost efficiency. It can be concluded that both optical and electrochemical techniques for the detection of CA-125 can be worthwhile, but each should be employed in specific circumstances to obtain the desired outcome more efficiently. In respect of the optical detection of CA-125, although this method is highly selective with a low LOD and can be employed for specific and real-time detection of CA-125, it is commonly expensive and hard to construct, which has restricted its clinical employment. On the other hand, electrochemical biosensors are highly sensitive, convenient to construct, cost-efficient, and recyclable, but they are mostly non-selective and at high currents, it is hard to control the sensing process. Therefore, these biosensors are more applicable for obtaining overall estimation when an exact result with high selectivity is not required.

## Figures and Tables

**Table 1:** Optical biosensors for CA-125 detection.

Method	Nanoplatform	Limit of Detection (LOD)	Linear Range	Ref
Fluorescent	Agarose nano-net	1.0 U/mL	0.05–1.45 U/mL	[36]
	Ag NPs	0.0018 U/mL	0.01–80 U/mL	[37]
	Ag NCs/GO or Ag/Au NCs/GO	1.26 ng/mL	2 ng/mL–6.7 µg/mL	[38]
	SPN/MIP or CNT/MIP	0.49 U/mL	3.12–150 U/mL	[39]
	rGO	50 mU/mL	50–2000 mU/mL	[40]
	3D CNT	10 pg/mL	10 pg/mL–1 μg/mL	[41]
	Magnetic NPs	0.26 U/mL	0–500 U/Ml	[39]
	NA	4 μg/mL	4–250 μg/mL	[40]
	Magnetic graphene oxide (GO/Fe_3_O_4_)	50 mU/mL	0.0005–40 U/mL	[41]
	Combination of NaYF_4_: Yb, Tm, and Ag NPs	120 U/mL	5–100 U/mL	[42]
	Ag NCs	0.015 U/mL	0.01–2 U/mL	[43]
	Au doped sol–gel matrix	1.45 U/mL	2–127 U/mL	[44]
FRET	PAMAM-dendrimer/Au NPs	0.5 fg/mL	1 fg/mL–1 ng/mL	[45]
	CuO NPs	3 × 10^−4^ ng/mL	2 × 10^−4^ ng/mL–100 U/mL	[46]
	CQDs	0.66 U/mL	0.01–129 U/mL	[47]
Photoluminescent	Ag_2_S QDs	0.07 ng/mL	0.1–106 ng/mL	[48]
CL	Graphene QDs	0.05 U/mL	0.1–600 U/mL	[49]
	SiO_2_ NPs	0.17 U/mL	0.5–400 U/mL	[50]
	NA	0.15 U/mL	0.50–80 U/mL	[51]
	MPs	2 U/mL	0–400 U/mL	[52]
ECL	Ru-Au NPs/GR	0.005 U/mL	0.01–100 U/mL	[53]
	Cd/Se NCs	5 × 10^−5^ U/mL	10^−4^–1 U/mL	[54]
	Dendrimer-sulfanilic acid-Ru(bpy)32+ and Dendrimer-CdTe@CdS nanocomposite	1.1 µU/mL	1 µU/mL–1 U/mL	[55]
	CdTe/CdS QDs	0.034 mU/mL	0.0001 U/mL–10 U/mL	[56]
	AgInS2/ZnS nanocrystals	1 × 10^−6^ U/mL	5 × 10^−6^–5 × 10^−3^ U/mL	[57]
	Au NPs	0.0074 U/mL	0.01–100 U/mL	[58]
	Amino-functionalized mesoporous silica NPs	4.3 mU/mL	0.01–50 U/mL	[59]
	Fe_3_O_4_	8.0 μU/mL	0–10 mU/mL	[60]
	Au-Ag nanocomposite-functionalized graphene	2.5 mU/mL	0.008–50 U/mL	[61]
	Fe_3_O_4_	0.4 mU/mL	0.001–5 U/mL	[62]
	Fe_3_O_4_	0.032 μU/mL	0.2–100 μU/mL	[63]
SPR	Au NPs	5 nM	0.25–9.0 μg/mL	[64]
	Au NPs	0.66 U/mL	2.2–150 U/mL	[65]
	Au-SPE film	0.1 U/mL	0.1–300 U/mL	[66]
	Au NPs	0.1 U/mL	0.1–40 U/mL	[67]
	Au/ZnO nanocomposite	0.025 U/mL	1–40 U/mL	[68]
SERS	Au NPs	NA	NA	[69]
	Ag NPs	NA	NA	[70]
Plasmon Resonance Scattering (PRS)	Au nanorods	0.4 U/mL	1–80 U/mL	[71]
Colorimetric	Ag/Au NPs	30 U/mL	0–1000 U/mL	[72]
	Hollow polydopamine-Au and Fe_3_O_4_ NPs	0.1 U/mL	0.1–100 U/mL	[73]

**Table 2:** Electrochemical biosensors for CA-125 detection.

Electrode Material	Coating Material	Advantages	Disadvantages	Features	Ref.
GCE	AuNPs	High sensitivity, low cost, short test time	Narrow linear range, detects lower-than-average biomarker values (35 U/mL)	Stabilizer: cellulose acetate membrane, cysteamine (CysA) sulfur-containing biomolecule	[97]
GCE	(Silver nanoparticles) Ag NPs	High electrical conductivity and biocompatibility, and low toxicity. Optical and thermal attributes, support for electrocatalytic activity	Aggregation of Ag with solvent evaporation causes gaps and leads to low conductivity		[97]
GCE	* Ag NPs with graphene quantum dot (Ag-DPA-GQDs ink)	Measures different concentrations of CA-125 biomarker		Conductivity: 290 mS [86] The linear range is 0.01–400. Descriptions: Ag-DPA-GQDs nano-ink deposition on GCE electrode	[97,98]
GCE	Antimonene quantum dots (AMQDs)	Reduces the cost of analysis	LOD is 4.4 μM.	Catalase for H_2_O_2_ reduction is immobilized on AMQDs for cyclic voltammetry and amperometry detection.	[99]
GCE	Nafion + MPBB antibody	Detects at low concentration, detects OC early and can be used to screen at-risk individuals.		The linear range is 5–50 ng/mL and 100–500 ng/mL	[100]
Three-dimensional gold electrode(Au/GNS/Ab-modified electrode)	Silicon nanoparticles (SiNPs)	Linked to the immunosensor CA-125, improved electrochemical performance.		The linear range is 1 fg/mL–1 μg/mL	[100,103]
GCE	Zinc oxide (ZnO)-based NP	High repeatability, specificity, and durability	Acceptable stability	Linear range is 2.5 ng/μL–1 ng/μL	[100]
	Graphene-polyaniline-based	Improves early-stage diagnosis		The linear range is 0.92 pg/L to 15.20 ng/L.	[100]
GCE	MOF-808/CNT	Biocompatible surface, high stability, electrochemically enhanced		The linear range is 0.001–30 ng/mL	[104]
Biotin-modified carbon paste electrodes	Au NPs	Stability, biological adaptability	Narrow linear range Detects lower-than-average biomarker levels (35 U/mL)		[97]

* Anti-CA-125 antibody was immobilized on Ag-DPA-GQDs and CysA-Au NPs. Biofluid was directed via a microchannel mounted on the sensor surface to detect CA-125 [97].

**Table 3:** Electrochemical detection methods.

Electrochemical Detection Methodology	Assay Strategy
Amperometric [98]	Nanostructured colloidal gold immunosensor Immunosensor based on multiwalled carbon nanotubes Tagging technique for redox probes Biosensor with a molecular imprint Magnetic bead immunosensor Immunosensor nanoparticle
A field-effect transistor (FET) [98]	Nanotube-based immunosensor Label-free immunosensor
Potentiometric [98]	Immunosensor-arrayed microfluidic device Magnetic bead immunosensor

**Table 5:** Comparison of the performance of a range of CA-125 commercial detection kits.

Commercial CA-125 Kits	Assay Sensitivity	Assay Range	Sample Type	Assay Time (h)
LifeSpan	-	1.563–100 U/mL	Plasma, serum	3.5
RayBiotech	0.6 U/mL	0.6–400 U/mL	Cell culture supernatants, plasma, serum	-
Aviva Systems	6.5 pg/mL	15.6–1000 pg/mL	Serum, plasma, tissue homogenates, and other biological fluids	3
Wuhan Fine	1.875 IU/mL	3.125–200 IU/mL	Serum, plasma, and other biological fluids	-
(DEMEDITEC Diagnostics GmbH)	0.25 U/mL	25–600 U/mL	Serum, plasma	1 h and 15 min
(Thermo Fisher Scientific)	-	0.55–400 U/mL	Plasma, 50 µL; serum, 50 µL; supernatant, 100 µL	4 h and 45 min
Novus Biologicals	3.8 U/mL	3.8 U/mL	Serum, plasma	-
